# Supplementary figures and images for: New retron systems from environmental bacteria identify triggers of anti-phage defense and expand tools for genome editing
Source: PLoS Biol. 2025 Oct 23;23(10):e3003042. doi: 10.1371/journal.pbio.3003042 (PMC12548924; doi:10.1371/journal.pbio.3003042)

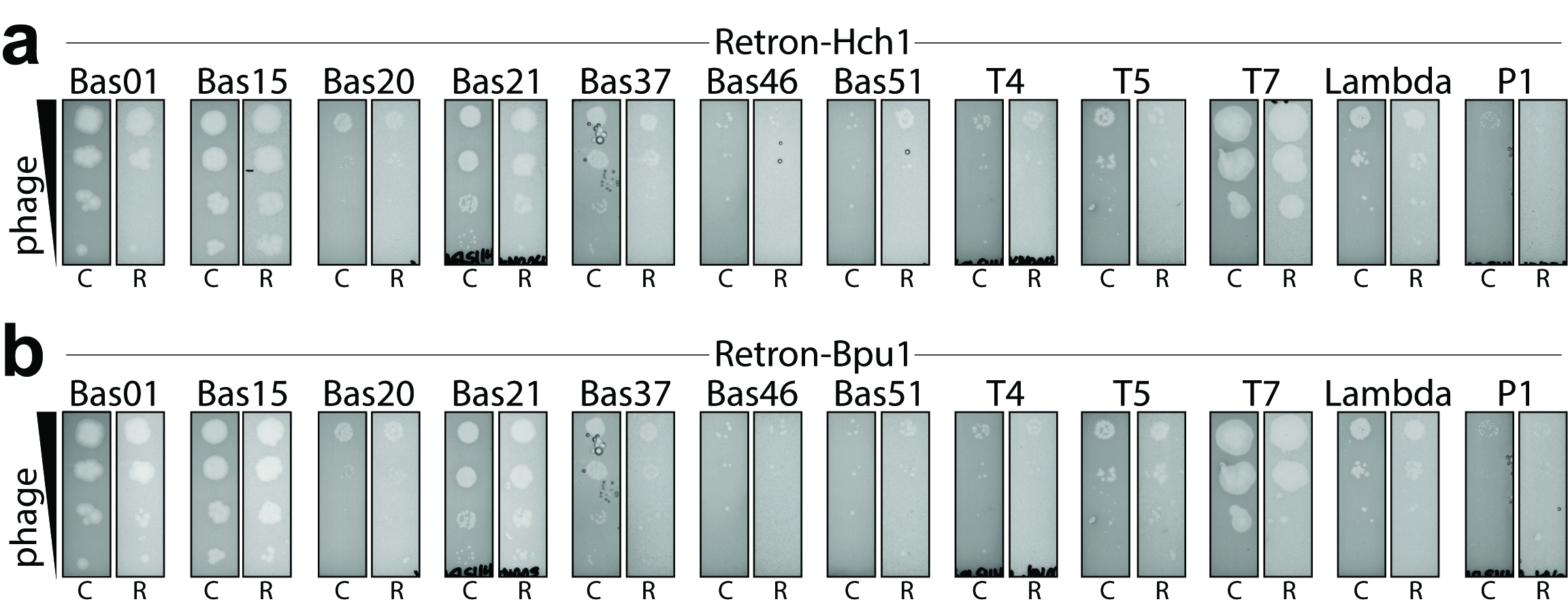

Supplement: S1 Fig — Images are matched phage plaque assays for each phage with C indicating the control condition and R indicating the retron-expressing condition. (TIF) [file pbio.3003042.s001.tif]

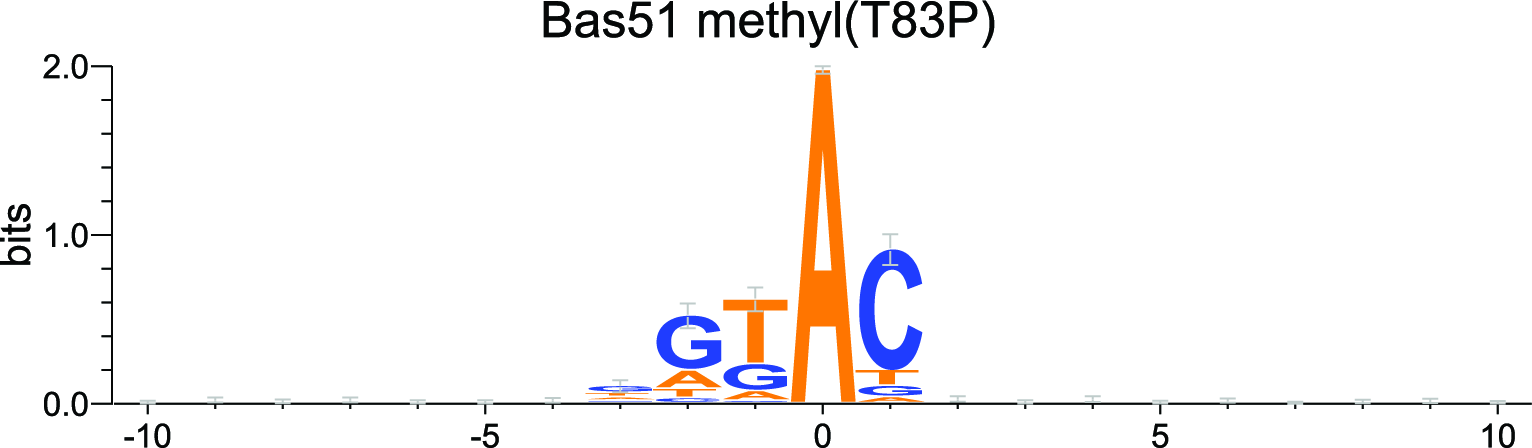

Supplement: S2 Fig — (TIF) [file pbio.3003042.s002.tif]

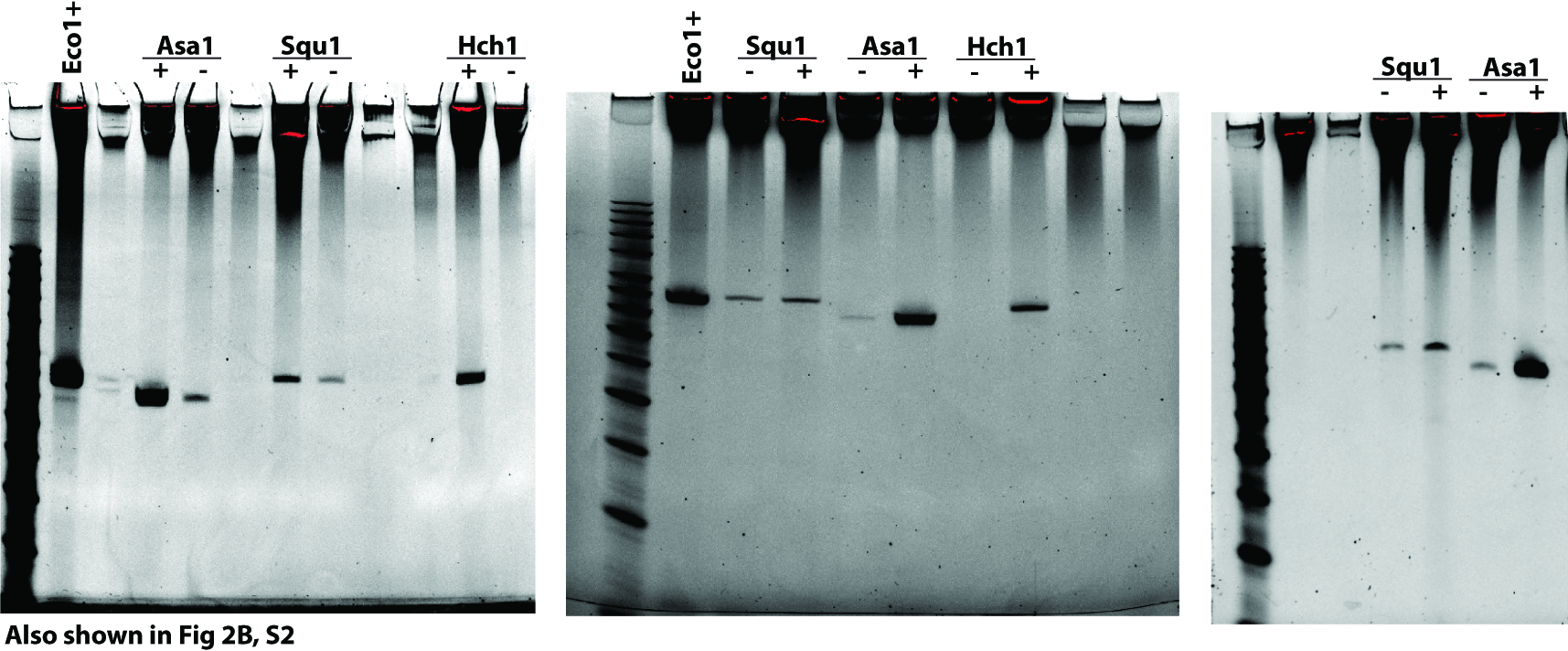

Supplement: S3 Fig — (TIF) [file pbio.3003042.s003.tif]

# Uncropped Gels

Figure 1b:

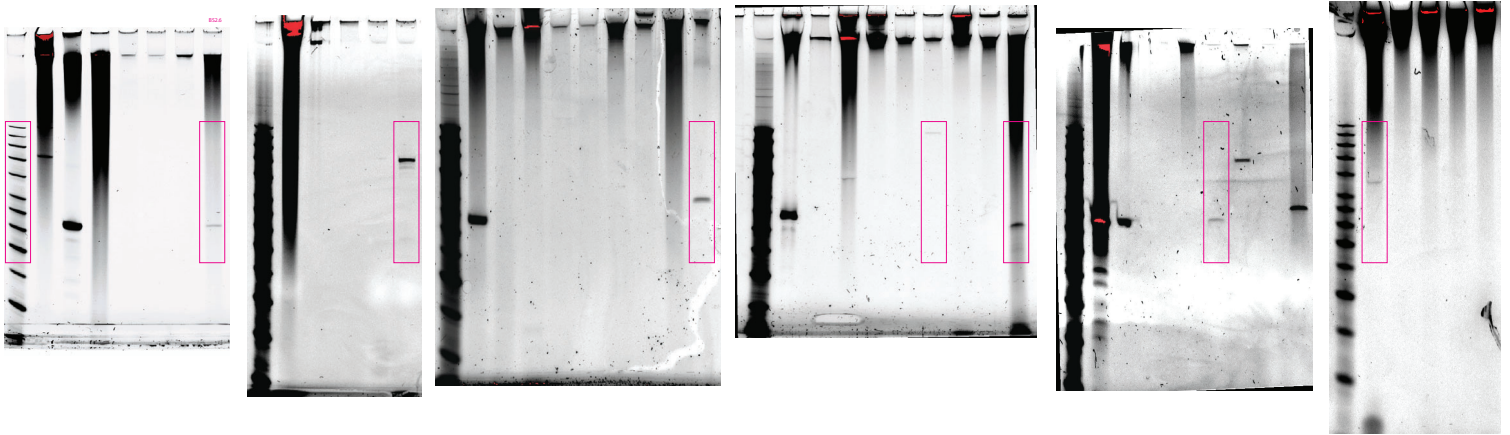

Figure 2b:

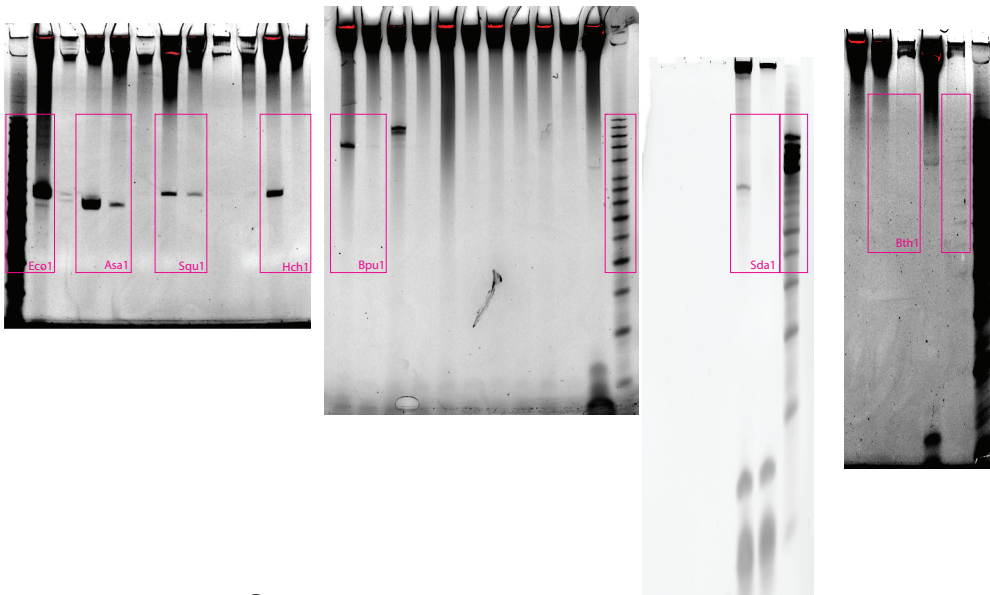

Figure 5f:

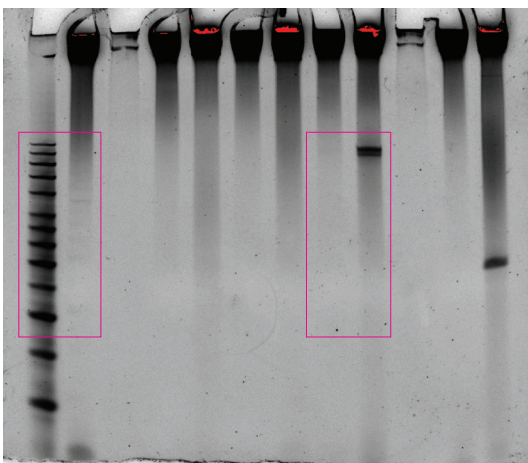

Supplement: S1 Raw Images — (PDF) [file pbio.3003042.s010.pdf]
