# Supplementary material for: New retron systems from environmental bacteria identify triggers of anti-phage defense and expand tools for genome editing
Source: PLoS Biol. 2025 Oct 23;23(10):e3003042. doi: 10.1371/journal.pbio.3003042 (PMC12548924; doi:10.1371/journal.pbio.3003042)
Supplement: S1 File — (HTML) [file pbio.3003042.s004.html]

Flagcsnap
