# Supplementary material for: New retron systems from environmental bacteria identify triggers of anti-phage defense and expand tools for genome editing
Source: PLoS Biol. 2025 Oct 23;23(10):e3003042. doi: 10.1371/journal.pbio.3003042 (PMC12548924; doi:10.1371/journal.pbio.3003042)
Supplement: Attachment — Submitted filename: 2025_Rebuttal_09_04.docx [file pbio.3003042.s013.docx]

We are grateful for the efforts of all reviewers during the consideration of our initial submission. The comments were constructive and we have made efforts to address them as fully as possible. We believe that this has resulted in an improved, revised manuscript. Below is a summary of newly added data, followed by a point-by-point response to each reviewer comment.

**Summary of New Experiments**

1. We uncovered additional mechanistic detail about the phage defense of retrons-Asa1 and –Squ1 against Bas51. Specifically, we were able to use nanopore sequencing of Bas51 to identify the methylation motif of the adenine methyltransferase that we had identified as the trigger of these retrons, and showed that this motif GATC is present in the RT-DNA of both sensitive retrons. **Moreover, we were able to extend this mechanism into the natural host of the Retron-Squ1**, showing that the methyltransferase leads to growth suppression of in the isolated *S. quinivorans* strain carrying the retron, but not a strain where the retron has been removed. Overexpression of the system in the natural host further exaggerates this effect. These new experiments are described in Figure 4e-j.

**Figure 4. *E)*** *Logo showing the predominant motif of methylated adenine sites in wild-type Bas51 phage.* ***F)*** *Stacked bar plot showing distribution of adenine methylation site motifs in wild-type Bas51 phage compared to a Bas51 methyltransferase mutant (T83P).* ***G)*** *Retron-Asa1 predicted ncRNA secondary structure with reverse transcribed region colored in orange and GATC motif colored in blue.* ***H)*** *Retron-Squ1 predicted ncRNA secondary structure with GATC motif colored in blue.* ***I)*** *Growth curves comparing OD_600_ of wild-type* S. quinivorans *transformed with an empty vector, wild-type* S. quinivorans *transformed with methyltransferase, and* S. quinivorans *ΔSqu1 transformed with methyltransferase over 8 hrs. Methyltransferase expression was induced at 2 hrs. Bar plot on right compares OD_600_ measurements of these samples at the 5 hr timepoint. Open circles show each of three biological replicates and the bar shows the mean*  *(One-way ANOVA P=0.0017; follow-up testing with Tukey’s, corrected for multiple comparisons, wt:empty vs wt:methyltransferase P=0.0045; wt:methyltransferase vs ΔSqu1:methyltransferase P=0.0021, all other conditions ns).* ***J)*** *Growth curves comparing OD_600_ of* S. quinivorans *ΔSqu1 transformed with either empty vector + methyltransferase, Retron-Squ1 + empty vector, or Retron-Squ1 + methyltransferase over 8 hrs. Methyltransferase expression was induced at 2 hrs. Bar plot on right compares OD_600_ measurements of these samples at the 5 hr timepoint. Open circles show each of three biological replicates and the bar shows the mean. (One-way ANOVA P<0.0001; follow-up testing with Tukey’s, corrected for multiple comparisons, empty&methyltransferase vs Squ1&methyltransferase P<0.0001; Squ1&empty vs Squ1&methyltransferase P<0.0001, all other conditions ns).*

2. With additional sequencing and analysis, we identified the likely source of the RT-DNA band that we had previously been unable to explain. We found a system that is carried on a plasmid in *Bacillus thuringiensis*. We initially missed this system because the retron RT is interrupted by a group II intron and its associated group II intron RT. We found upon transferring the system to *E. coli* that some RT-DNA is produced even with the interrupting group II intron, but if we remove that intron the abundance of RT-DNA increases substantially. These findings are described in revised Figures 1m and 5f.

**Figure 1m.** Newly identified retron operon.

**Figure 5. *F)*** *RT-DNA production from PAGE analyses of Retron-Bth1 in its native host and in E. coli with the gII intron deleted, respectively (uncropped gel in Supplementary Figure 5).*

3. In the original manuscript, we identified a retron where the typical effector appeared to have been replaced with elements of a different defense system, but lacked any functional data to disentangle the components. We have now included new tests of phage defense when deleting each component. We find that the non-retron components are able to provide defense as a standalone system, whereas we did not find evidence for defense from the retron components. These new experiments are described in Figure 5e.

**Figure 5. *E)*** *Phage defense (log transformed) calculated as the pfu (plaque forming units) per ml on a control strain not expressing the retron divided by pfu per ml on the retron expressing strain, for Retron-Sda1 against Bas51.. Increasing numbers indicate defense phenotypes. For each variant, open circles show each of three biological replicates and the closed circle shows the mean. One-way ANOVA P=0.002, Dunnett’s follow-up testing (corrected for multiple comparisons) empty plasmid versus wt (P=0.0031), ΔHTH (P=0.0037), ΔncRNA (P=0.0009), dRT (P=0.0006), all other conditions ns. Variants are explained by schematic in the middle with example plaque assays to the right for each variant. Deletion of DUF3037 created a toxic construct where phage defense could not be quantified.*

4. We expanded our panel of phages tested from 4 to 12, finding additional defense phenotypes for retrons-Asa1 and –Squ1. We also tested for defense with overexpression of the retrons that showed no defense when uninduced, but still found no defense phenotypes for those two retrons. These experiments are described in Figure 3 and Supplementary Figure 3.

**Figure 3.** A) Phage defense (log transformed) was calculated as the pfu (plaque forming units) per ml on a control strain not expressing the retron divided by pfu per ml on the retron expressing strain. Increasing numbers indicate defense phenotypes. Three biological replicates are shown as open circles and the mean is shown as a closed circle. Retron-Asa1 exhibits defense (one-way ANOVA P<0.0001) against Bas01 (P<0.0038), Bas20 (P<0.0001), Bas21 (P<0.0141), and Bas51 (P<0.0381). Individual phage statistics calculated by Dunnett’s test corrected for multiple comparisons. B) Phage defense shown exactly as in A, but for Retron-Squ1, which exhibits defense (one-way ANOVA P<0.0001) against Bas01 (P<0.0001), Bas15 (P<0.0001), Bas20 (P<0.0001), Bas21 (P<0.0001), Bas51 (P<0.0001), and T5 (P=0.0001). Individual phage statistics calculated by Dunnett’s test corrected for multiple comparisons. C) Phage defense shown exactly as in A, but for Retron-Hch1, which does not exhibit defense against this panel of phages (one-way ANOVA P=0.7125). D) Phage defense shown exactly as in A, but for Retron-Bpu1, which does not exhibit defense against this panel of phages (one-way ANOVA P=0.0644).

**Supplementary Figure 3.** Phage defense testing of retrons-Hch1 (A) and –Bpu1 (B) in bSLS.114 with overexpression. Images are matched phage plaque assays for each phage with C indicating the control condition and R indicating the retron-expressing condition.

**Editorial**

IMPORTANT: After discussion with the Academic Editor and the cross-comments, you should aim to address all of the textual/formatting changes recommended by the reviewers. In addition, we will expect further experimental validation of their retron systems, as suggested by reviewers 2 and 3. However, we do not expect you to complete the genome editing in a non-E.coli background as this could be highly challenging, and might be outside of the scope of the study.

-----------------------------------------
REVIEWERS' COMMENTS:
-----------------------------------------

**Reviewer #1:**

*It was a pleasure to review this creative study by Nakamura and colleagues on the characterization of retron elements from environmental bacteria. Their unbiased sampling approach impressively demonstrates the abundance of these systems, and the in vivo identification is a rare and valuable contribution to the phage defense field, which has largely focused on in silico identification. This is particularly important as it allows for the study of defense systems in their native host backgrounds, a significant limitation of most current research. The manuscript is well written, and the data and figures are of high quality.*

Thank you!

*I have the following suggestions for improvement:

Even though the gene color scheme is labelled in 1d, i.e. pink arrow = effector, it would be helpful to clearly state this scheme at end of the legend. It took me two passes to understand the significance of the colors. Additionally, the abbreviations used in the functional annotations should be expanded in the legend, e.g. WH, PRTase, RT etc. It is unclear to me which of these are the DNA-binding domain containing proteins mentioned in the following paragraph.*

Apologies. We have added explicit descriptions of the operon components and colors as well as a written out all abbreviations in the legend, including identifying the WH DNA-binding domain.

Again in Figure 1E, why is the DUF3037 gene colored as an effector? Do the authors hypothesize this protein acts as the effector in this system and what is the evidence?

Great point, and even more important given new data that we added showing that the HipA and DUF appear to be a standalone defense system, rather than part of the retron. That new data is included in Figure 5 and we have colored the DUF3037 grey in Figure 1 to indicate that it is not part of the retron system.

Text beginning at paragraph "Retron-Sda1 is an example…" goes immediately into retron classifications. For the non-expert reader, it would be helpful to include some text in the introduction to contextualize the classification of retron systems i.e. how many are there are what are the classification criteria? There is some text related to this in the fourth paragraph of the discussion, but it came too late to help me contextualize the data in Figure 1 and associated paragraph.

We apologize and have added a statement much earlier in the manuscript, “Retrons have been classified into 13 subtypes based on effector type and operon structure.”

More information should be given on how PDC-M66 was classified as a putative defense system classification (in the second paragraph following Figure 1). What criteria was used to make this prediction? I could not find any publications mentioning PDC-M66.

Apologies for the confusion. PDC-M66 is the designation given by PADLOC to this putative defense system, where PDC stands for phage defense candidate. What this indicates is that there is a class of similar putative defense systems found across the identified tree. We have clarified the language in the text.

We go on to make the link to an experimentally-validated system called DS-6. In this revised version, we also go further, adding experimental data to show that the two protein HipA-like/DUF3037 is capable of standalone phage defense.

**Figure 5. *E)*** *Phage defense (log transformed) calculated as the pfu (plaque forming units) per ml on a control strain not expressing the retron divided by pfu per ml on the retron expressing strain, for Retron-Sda1 against Bas51.. Increasing numbers indicate defense phenotypes. For each variant, open circles show each of three biological replicates and the closed circle shows the mean. One-way ANOVA P=0.002, Dunnett’s follow-up testing (corrected for multiple comparisons) empty plasmid versus wt (P=0.0031), ΔHTH (P=0.0037), ΔncRNA (P=0.0009), dRT (P=0.0006), all other conditions ns. Variants are explained by schematic in the middle with example plaque assays to the right for each variant. Deletion of DUF3037 created a toxic construct where phage defense could not be quantified.*

In the third paragraph after Figure 3 "However, the Asa1 retron showed a subtle phenotype…". Should this say Squ1 as it appears from the figure?

This paragraph has been rewritten to reflect the larger phage defense panel and this error has been removed in the process.

As Bas51 is not a well known coliphage, it would be helpful to give some more information about this phage to contextualize its sensitivity to Asa1 and Squ1. Is it related to any of the other phages used? I.e. does it share any similarity with phage T5?

Apologies, we have included additional information on our expanded panel of phages:

To better understand how naturally sourced retrons function as defense systems, we next tested the four retrons with intact, type-identified operons for defense against a panel of twelve E. coli phages, including examples of Drexlerviridae (Bas01), Siphoviridae (Bas15, Bas20, Bas21), Demerecviridae (T5), Myoviridae (Bas37, T4, P1), Mosigvirus (Bas46), Vequintavirinae (Bas51), Autographiviridae (T7), and Siphoviridae (lambda)^30^.

I found the second paragraph of the section titled "Defense Mechanisms of Retron-Asa1 and -Squ1" hard to follow. I infer that 10 escape phages were sequenced in total but only 6 were found to be unique after polynucleotide and synonymous changes were ignored. If this is correct, please add a sentence stating this clearly. A symbol next to the tracks in figure 5 a&b could be added to indicate escapees found to have redundant mutations.

We appreciate this chance to fix an error. We actually sequenced 8 total escapees, not 10, and show the mutations for all 8 in panels 5a and 5b. We have updated the text to be clearer about the escapee mutations we found.

We used nanopore sequencing to identify potential trigger genes in mutant phage escapees, sequencing four escapees of Retron-Asa1 and four of Retron-Squ1. When analyzing data, we ignored mutations in polynucleotide stretches (known to be enriched for nanopore errors) and synonymous mutations. All escapees that we sequenced contained mutations in an adenine methyltransferase gene, with five distinct mutations across the eight escapee phages (Fig 4a). Additionally, four of eight contained an identical premature stop mutation in a glycosidase gene (Fig 4b).

No data is cited for the statement "Retron-Asa1 recombitron's editing rates are likely partly a result of its RT-DNA production in E. coli…". Does this reference figure 3B? Can the abundance of RT-DNA by Asa1 be reproduced in biological replicates?

Yes, retron-Asa1 does consistently produce more RT-DNA than the other retrons tested for editing when we assessed in parallel experiments. We have added additional replicate gels as Supplemental Figure 6 to illustrate this relative abundance. However, we also softened the statement to simply point out this correlation rather, “Interestingly, Retron-Asa1, which displayed the highest editing rate, and also consistently produced more abundant RT-DNA when compared to retrons with lower editing rates (Supplemental Figure 6).”

There is a citation missing for the statement "the majority do and should be identifiable when screening for RT-DNA bands."

We have removed this statement in the revised manuscript.

I found the following typographical errors:

"retrons have also be repurposed"

Fixed, thank you.

Figure three legend "E. Coli" (change to lowercase c)

Fixed, thank you.

-----------------------------------------

**Reviewer #2:**

The study by Nakamura, Zhang, et al. isolates new retrons from natural bacterial isolates and includes a basic characterization of some of these in bacterial immunity and genome editing. I have great sympathy for this work, but am unhappy about its currently very streamlined style that lacks a variety of content and explanations which would make it more convincing and accessible to non-experts. Most importantly, the two most interesting newly found retrons are only briefly mentioned but not at all explored or validated in wet lab experiments. This is very confusing.

We have added new experiments to better investigate the two strangest retrons we found, retron-Sda1 and retron-Bth1. Interestingly, both of these retrons appear to be disrupted by a intervening gene or genes, and both appear to have lost their effector (and thus phage defense potential). We found that retron-Sda1 has been disrupted by a standalone HipA-DUF defense system, which is active on its own and defends against phage Bas51. However, we found no phage defense for the retron components. Retron-Bth1 is disrupted by a group II intron. Although it surprisingly produces some RT-DNA despite this disruption, the abundance of RT-DNA is dramatically increased by removing the group II intron.

Fig 1. A) Schematic of the isolation of bacteria from soil and water samples and identification of possible retron-bearing hosts using PAGE analysis. B) Retron RT-DNA bands from PAGE analyses (uncropped gels included as Supplementary Figure 1) . C) Overview of sampling, screening of colonies, and identification of retron hosts. D) Hosts displaying an RT-DNA band were subjected to whole-genome sequencing. E) RT-DNA was subjected to unbiased adapter addition and sequencing. F) Schematic of canonical retron operon with components annotated, effector in pink, non-coding RNA (ncRNA) in orange, and reverse transcriptase in purple. F-M) Seven putative retron operons, identifying the retron subtype, host species, and predicted two-dimensional structure of the ncRNA. For retrons where RT-DNA sequencing is available, sequencing coverage is plotted onto ncRNA in an orange heatmap (heatmap legend indicated number of sequencing reads mapped). Gene abbreviations: NDT (nucleoside deoxyribosyltransferase-like); PRTase (phosphoribosyltransferase); WH (winged helix-turn-helix DNA binding); HTH (helix-turn-helix domain containing); HipA (HipA-like); DUF (DUF3037 domain-containing protein).

Fig 5. E) Phage defense (log transformed) calculated as the pfu (plaque forming units) per ml on a control strain not expressing the retron divided by pfu per ml on the retron expressing strain, for Retron-Sda1 against Bas51.. Increasing numbers indicate defense phenotypes. For each variant, open circles show each of three biological replicates and the closed circle shows the mean. One-way ANOVA P=0.002, Dunnett’s follow-up testing (corrected for multiple comparisons) empty plasmid versus wt (P=0.0031), ΔHTH (P=0.0037), ΔncRNA (P=0.0009), dRT (P=0.0006), all other conditions ns. Variants are explained by schematic in the middle with example plaque assays to the right for each variant. Deletion of DUF3037 created a toxic construct where phage defense could not be quantified. F) RT-DNA production from PAGE analyses of Retron-Bth1 in its native host and in E. coli with the gII intron deleted, respectively (uncropped gel in Supplementary Figure 5).

The introduction is comparably short and completely focused on retrons as phage defense elements (and their research history) without any general context. My view is that this introduction may be appropriate for a specialist audience but should be widened to make the manuscript accessible also to readers from outside the field of bacterial immunity. I also missed a convincing argument for the relevance of this work beyond phage defense aficionados. The manuscript essentially says (third paragraph) that only retrons from a couple of hosts have been studied so far, but it fails to highlight why readers from outside the field should care about the characterization of additional ones. Of course there are various good reasons for this (e.g., the acquisition of better tools for molecular genetics) but I feel that this should be spelled out.

We have added additional text to the introduction:

Despite this, only a handful of retrons have been studied in the context of their natural host, and retron defense against phages has only been studied in E. coli^3,23-25^, B. subtilis^26^, and S. enterica^1^. Identifying a wider range of natural hosts would enable researchers to determine whether mechanisms identified biochemically or within model systems like E. coli are an accurate description of the biology. Furthermore, prior studies have been largely limited to retrons from clinical and laboratory strains of bacteria with few environmental examples (e.g. Vibrio mimicus^27^). Given the limited selection of experimentally confirmed retrons, characterization of additional retrons sourced from different environments could lead to the discovery of new defense mechanisms and the development of better biotechnology for application in molecular genetics.

The authors began their study with the isolation of retron-carrying hosts from natural samples based on the cultivation of environmental microbes and the subsequent screening for characteristic ssDNA bands on polyacrylamide gels. I do not doubt that this approach gave the authors some new retrons. However, the methodology of how they got there seems rather crude and very arbitrary in the sense that the procedure and possible limitations are not explained or justified in any way (also not in the discussion section). As an example, the authors used rich LB medium at 37°C for the isolation of bacteria from mostly nutrient-poor, ambient-temperature environments. This does not seem very intuitive to me and could greatly bias the selection towards very few highly resilient, fast-growing organisms out of the vast diversity of environmental microbes.

We absolutely acknowledge that our culturing conditions select for faster growing organisms and differ from the natural environments of these bacteria. For purely practical reasons we choose to focus on bacteria growing at 37 degrees in LB for ease as we went out in search of strains in the environment encoding retrons for the first time ever. We plan to build on this in years to come, and also hope that our approach of screening for RT-DNA bands inspires others to apply it to bacteria that grow at other temperatures and in other media. We have added this point to our revised discussion:

For purely practical reasons, we chose to grow bacteria mainly at 37° C in LB media in this study. These culture conditions undoubtedly select for faster growing organisms and differ from the natural environments from where we sourced these bacteria. Indeed, the overrepresentation of type II-A and III-A retrons may reflect selection due to culturing conditions favoring bacteria harboring these subtypes. Future work that tests a wider variety of culture conditions, including those that mimic natural environments, may find additional interesting retron-bearing bacteria and we hope that this work provides a roadmap for isolation of many new retron hosts from the wild.

I really liked the idea to screen for retron-expressing microbes using polyacrylamide gels because this opens the door to finding completely new, exotic kinds of retrons. However, the authors later largely relied on the standard tool PADLOC to identify the retrons in their isolates' genomes. While they could occasionally refine the PADLOC annotation using their experimental data, this makes the reader wonder why one should at all isolate environmental bacteria one by one to find new retrons. This would be much easier by simply putting hundreds of new metagenomic microbial genomes into PADLOC to get their retron loci (which could then be synthesized and studied). None of these important considerations are made anywhere in the study. Furthermore, the potentially most interesting discovery of this study - a non-identified organism (apparently isolated twice) which shows a retron-like band on gel but for which PADLOC found no retron - is only briefly mentioned but not discussed or explored at all. This organism may encode a completely new type of retron, and I think that this should be explored further. If there are clear RT-DNA bands visible on polyacrylamide gels (as highlighted by the authors), it would be easily possible to isolate and sequence these DNA molecules in order to map them back onto the genome.

Indeed, as discussed in response to earlier comments, we found the missing retron! It is carried on extrachromosomal DNA (quite possibly a jumbophage, but could be a plasmid) rather than the bacterial chromosome, which would have made it harder to identify and match to a host species from metagenomics sequences. More importantly, because the RT gene is disrupted by the insertion of a group II intron it is not readily identified as a retron by annotation tools. We never would have identified this odd, disrupted, but still DNA-producing retron if we had not found the RT-DNA in its natural host and gone searching deeply for RTs in the genome. In addition to presenting these new results, we have amended the discussion as follows:

PADLOC identified retron operons in whole genome sequencing data of most samples where clear, putative RT-DNA bands were visible on polyacrylamide gels. However, the case of retron-Bth1 illustrates a more unique example where the physical identification of RT-DNA led us to describe a piece of biology that would have likely been overlooked in metagenomics. First, it is found on an extrachromosomal element and the fact that we had the strain let us link this element to the species. Second, because the RT gene is disrupted by the insertion of a group II intron, is located in a clade of phage-encoded retrons, and is not associated with a canonical effector, it is not readily identified as a retron by annotation tools. We would likely have not identified this odd, disrupted, but still DNA-producing retron, if we had not found the RT-DNA in its natural host and gone searching deeply for RTs in the genome.

From their natural samples, the authors found six different retrons in cultivated organisms which they studied further following standard procedures in the field. Initially, they focused on one specific retron (Retron-Sda1) which they analyzed bioinformatically because it seems to encode a different potential effector of abortive infection compared to what would be common for this type of retrons (type III). This is in principle interesting. However, the authors then specifically exclude this exact retron from any wet lab experiments testing ssDNA production in E. coli or bacterial immunity. This is not explained in any way beyond the authors' puzzling statement that "We also did not further study the III-A1-like Retron-Sda1".

We have now added much more experimental data on retron-Sda1. Please see response to Reviewer #2’s first comment above.

After the bioinformatic analysis of Retron-Sda1 (without wet lab validation), the authors cloned their other new retron variants to show biological activity using ectopic expression in the model organism E. coli. Possible limitations of this host for the diverse retrons from very different other bacteria are neither explored nor mentioned anywhere. Not unsurprisingly, enforced expression of these retrons using T7 RNA polymerase in BL21-AI results in detectable retron DNA production (Figure 3). The gel also shows the well-characterized Eco1 retron - possibly as a control - which is neither mentioned nor explained in this context (but pops up again later in a different context with some more explanations).

Apologies, we have now included a reference in the text to the fact that we use Retron-Eco1 as a control for RT-DNA production in E. coli.

Subsequently, the authors used the same plasmids to test a possible activity of their new retrons against four different E. coli phages. I was very confused by these experiments because their model organism - E. coli K-12 MG1655 - does not encode the T7 RNA polymerase that they used in the BL21-AI strain to overexpress the retron loci. Did the authors now rely on the natural promoters of these loci, and if yes, is there any evidence that these may be functional / expressed in E. coli?

Apologies, we should have been clearer on this point. Most of the retrons displayed RT-DNA production even with no T7 overexpression, just using their natural promoters, which were cloned into E. coli along with the operon. We prefer to characterize phage defense without overexpression because the overexpression can slow bacterial growth substantially, making quantification of phage defense difficult. We have clarified this in the text.

For this test, cultures of E. coli MG1655 were transformed with Retrons-Asa1, -Squ1, -Hch1, and -Bpu1 (same plasmids as Fig 3), using the endogenous promoters to drive retron expression.

That said, we did not find phage defense for two retrons that produce very little RT-DNA when uninduced, retrons-Hch1 and –Bpu1 (retrons-Sda1 and –Bth1 are odd and discussed at length elsewhere). We retested phage defense in BL21-AI with induction using these two retrons with an expanded panel of phages, but still found no defense phenotypes.

**Supplementary Figure 3.** Phage defense testing of retrons-Hch1 (A) and –Bpu1 (B) in bSLS.114 with overexpression. Images are matched phage plaque assays for each phage with C indicating the control condition and R indicating the retron-expressing condition.

From their data, the authors claim that one of the retrons, Asa1, causes smaller plaques of phage T5 (which would be clear evidence of bacterial immunity) but do not provide a quantification of plaque size - this needs to be added (possibly also for Squ1 where at least on the image the effect looks much stronger). A suggested "strong trend of defense" of Asa1 against phage Bas51 seems to be largely due to two replicate experiments (Figure 4G), though the effect on plaque formation (Figure 4H) is quite clear. Evidently, the Squ1 retron defends against phage Bas51 by strongly reducing plaque counts.

We repeated the phage defense assays with the four intact retrons that could be expressed in E. coli, additionally using a wider range of phages. We find that retrons-Asa1 and –Squ1 both defend against multiple phages as assessed by plaque formation and prefer to remove any reference to trends or plaque size phenotypes, which are difficult to quantify objectively.

**Figure 3.** A) Phage defense (log transformed) was calculated as the pfu (plaque forming units) per ml on a control strain not expressing the retron divided by pfu per ml on the retron expressing strain. Increasing numbers indicate defense phenotypes. Three biological replicates are shown as open circles and the mean is shown as a closed circle. Retron-Asa1 exhibits defense (one-way ANOVA P<0.0001) against Bas01 (P<0.0038), Bas20 (P<0.0001), Bas21 (P<0.0141), and Bas51 (P<0.0381). Individual phage statistics calculated by Dunnett’s test corrected for multiple comparisons. B) Phage defense shown exactly as in A, but for Retron-Squ1, which exhibits defense (one-way ANOVA P<0.0001) against Bas01 (P<0.0001), Bas15 (P<0.0001), Bas20 (P<0.0001), Bas21 (P<0.0001), Bas51 (P<0.0001), and T5 (P=0.0001). Individual phage statistics calculated by Dunnett’s test corrected for multiple comparisons. C) Phage defense shown exactly as in A, but for Retron-Hch1, which does not exhibit defense against this panel of phages (one-way ANOVA P=0.7125). D) Phage defense shown exactly as in A, but for Retron-Bpu1, which does not exhibit defense against this panel of phages (one-way ANOVA P=0.0644).PFU/mL of T4 phage on strains containing constructs with naturally sourced retrons (Kruskal-Wallis test P=0.3385). B) Titration of T4 on strains containing constructs with naturally sourced retrons. C) PFU/mL of T5 phage on strains containing constructs with naturally sourced retrons (Kruskal-Wallis test P=0.0963). D) Titration of T5 on strains containing constructs with naturally sourced retrons. E) PFU/mL of lambda phage on strains containing constructs with naturally sourced retrons (Kruskal-Wallis test P=0.0984). F) Titration of lambda phage on strains containing constructs with naturally sourced retrons.. G) PFU/mL of Bas51 phage on strains containing constructs with naturally sourced retrons (Kruskal-Wallis test P=0.0047, follow-up Dunn’s test corrected for multiple comparisons: C16.5 vs no retron P=0.0631, BS2.6 vs no retron P=0.0009).

Following standard procedures in the field, the authors then isolate immunity-resistant phage mutants to identify phage genes in Bas51 which may encode triggers of Asa1 and Squ1 retron immunity or interact with retron immunity in another way. For both retrons, the authors isolated phages with mutations in both an N6 adenine methyltransferase and a glycosidase gene. It is implied (but not shown or discussed) that these mutations may cause full or partial loss of function. Using targeted mutations in the phage genome, the authors convincingly argue that the methyltransferase (likely by modifying the retron DNA, though this is not shown) may trigger retron immunity while the glycosidase might have some other role which is not explored in any way.

We have added substantial new mechanistic detail about the methyltransferase. First, we used the kinetics of nanopore sequencing on the Bas51 phage genome to map adenine methylation, finding the strongest motif to be GATC. Introducing the escapee mutation removes methylation at GATC sites, showing that the mutation is in fact a loss of function at that motif. Finally, we show that both of the retron triggered by Bas51 contain GATC motifs in their RT-DNA. We believe that this fully explains the triggering and that the glycosidase is probably not a direct trigger, but since we found the mutations and a slight phenotype when we introduced the mutation, we leave the data in for readers to interpret.

Fig 4. E) Logo showing the predominant motif of methylated adenine sites in wild-type Bas51 phage. F) Stacked bar plot showing distribution of adenine methylation site motifs in wild-type Bas51 phage compared to a Bas51 methyltransferase mutant (T83P). G) Retron-Asa1 predicted ncRNA secondary structure with reverse transcribed region colored in orange and GATC motif colored in blue. H) Retron-Squ1 predicted ncRNA secondary structure with GATC motif colored in blue.

To push forward, we also investigated the biology of this retron within the natural host of Retron-Squ1, *Serratia quinivorans*. We obtained genetic access to this host and knocked out the retron using lambda red recombineering. We then expressed the methyltransferase trigger within either the wild-type or knockout natural host. We find that it reduces growth (consistent with an abortive system) in the wildtype, but not the knockout host. This effect is exaggerated with overexpression of the system in the natural host.

**Figure 4.** I) Growth curves comparing OD600 of wild-type S. quinivorans transformed with an empty vector, wild-type S. quinivorans transformed with methyltransferase, and S. quinivorans ΔSqu1 transformed with methyltransferase over 8 hrs. Methyltransferase expression was induced at 2 hrs. Bar plot on right compares OD600 measurements of these samples at the 5 hr timepoint. Open circles show each of three biological replicates and the bar shows the mean (One-way ANOVA P=0.0017; follow-up testing with Tukey’s, corrected for multiple comparisons, wt:empty vs wt:methyltransferase P=0.0045; wt:methyltransferase vs ΔSqu1:methyltransferase P=0.0021, all other conditions ns). J) Growth curves comparing OD600 of S. quinivorans ΔSqu1 transformed with either empty vector + methyltransferase, Retron-Squ1 + empty vector, or Retron-Squ1 + methyltransferase over 8 hrs. Methyltransferase expression was induced at 2 hrs. Bar plot on right compares OD600 measurements of these samples at the 5 hr timepoint. Open circles show each of three biological replicates and the bar shows the mean. (One-way ANOVA P<0.0001; follow-up testing with Tukey’s, corrected for multiple comparisons, empty&methyltransferase vs Squ1&methyltransferase P<0.0001; Squ1&empty vs Squ1&methyltransferase P<0.0001, all other conditions ns).

Finally the authors used a LacZ-based assay to quantify the utility of their new retrons in bacterial genome editing compared to the previously characterized Eco1 retron. Confusingly, the authors did not use chromogenic LacZ substrates (e.g., "blue-white-selection" with X-Gal) but rather Illumina sequencing to quantify genome editing efficiency. Convincingly, the authors show that most new retrons have some detectable activity in genome editing though they are not as efficient as the Eco1 retron.

Apologies for this confusion. We should not have included the blue-white schematic since we don’t use it for quantification (we find Illumina amplicon sequencing to be much more reliable than color determination). We have removed the schematic and added “Quantified by Illumina Sequencing” to the Figure panel.

The discussion section is interesting and takes up some loose ends from this study, but it is (like the introduction) totally focused on the bacterial immunity function of the new retrons or their possible application in genome editing. Other relevant aspects - most importantly, specific limitations of this study - are not outlined or discussed.

We have added text discussing limitations of the study based on a point above about culture conditions and a point below about codon optimization.

Other points:
- future versions of the manuscript should have line numbers and page numbers to facilitate peer review

We have added page and line numbers.

- S. quinivorans and H. chinensis are mentioned totally out of the blue and as an abbreviation (end of first paragraph of the results section) without having been introduced before with full name

Apologies, these are now identified with their full name at first mention.

- important things like controls, annotated size markers, etc. are missing from the polyacrylamide gels shown in this study (and the other experiments also have a quite minimalist setup)

Our apologies, all gels now have markers annotating the ladder and we have increased annotation throughout.

- the manuscript uses a lot of jargon that is likely incomprehensible to non-experts if not explained ("PRTase", "NDT", etc.)

We regret this oversight, and now those gene abbreviations and others are expanded.

Gene abbreviations: NDT (nucleoside deoxyribosyltransferase-like); PRTase (phosphoribosyltransferase); WH (winged helix-turn-helix DNA binding); HTH (helix-turn-helix domain containing); HipA (HipA-like); DUF (DUF3037 domain-containing protein).

- the manuscript contains a variety of claims that I feel should be referenced but are not (e.g., "retrons often provide defense against phages when ported into a new species as well")

We should have cited supporting literature (e.g. Millman et al. *Cell* 2020, Bobonis et al., *Nature* 2022) for this statement, our apologies. However, we have removed this sentence while revising the text.

- unless I overlooked it, the paper does not mention that the ORFs in the retron loci have been codon-optimized when cloned for analysis in E. coli; this is obviously important and (if it has not been done) either has to be tested or mentioned as a critical limitation

No codon optimization was performed. In every case, we cloned the operon directly from the host. Every system had functional protein elements when in *E. coli* (based on RT-DNA production, phage defense, toxicity), so we know that these codons are capable of producing proteins, and our own experience with codon optimization when working across bacterial species has led us to prefer maintaining the natural nucleotide sequence. We have added clarification and discussion of this as a potential limitation to the revised manuscript:

We additionally used the natural codons for all testing, including for expression in *E. coli*. We found RT-DNA production for six of the seven systems, with the last system being toxic to cells, indicating that proteins are being produced in all cases. However, it is possible that optimizing codon usage could have increased the potency of some systems, which we did not test.

- the figure captions and the annotations of content in the figures are often very minimalist and leave the reader a bit puzzled (e.g., Figure S1)

In S1, we provide the uncropped gels that were cropped to produce Figure 1c as required by the journal. We have expanded the legend to indicate that the pink boxes are the cropped portions used in the main figure.

-----------------------------------------

**Reviewer #3 (Ilya Osterman):**

Review of the Manuscript on Novel Retrons in Bacterial Immune Defense and Genome Editing

Overall Assessment:
The manuscript presents a well-structured and engaging study on newly discovered retrons and their roles in bacterial immunity and genome editing. The work is highly relevant to the fields of microbiology, bacterial immunity, and biotechnology. The study provides valuable insights into the diversity of retrons, their defensive potential, and their biotechnological applications.

Thank you!

However, while the findings are intriguing, additional experiments are required to fully validate the claims, particularly in demonstrating the genome-editing potential of these retrons beyond E. coli and in expanding the analysis of their defensive capacity.

We appreciate this analysis and have substantially expanded the analysis of defense (see detailed comments below). Following Editorial evaluation, we were instructed that using the retrons to edit beyond *E. coli* would be outside the scope of this work.

Major Concerns:

Genome Editing in Diverse Hosts:

The authors successfully demonstrate genome editing using these retrons in E. coli, which aligns with previous studies on retron-based genome engineering.

However, since the retrons were isolated from a variety of bacterial species, it would significantly strengthen the manuscript if the authors could show their potential for genome editing in their native hosts or in a broader range of bacterial species. For instance, demonstrating editing capabilities in Bacillus, Serratia, Pseudomonas, or other less commonly studied hosts mentioned in the text would enhance the novelty of the study.

Expanding the experimental scope to non-E. coli species would provide compelling evidence of the broader applicability of these retrons as genome-editing tools.

Following Editorial evaluation, we were instructed that using the retrons to edit beyond *E. coli* would be outside the scope of this work.

Phage Defense Potential:

The manuscript presents data on the ability of these retrons to protect E. coli against a panel of four phages.

While this is an important first step, testing these retrons against a larger panel of phages would provide more robust evidence of their defensive potential. A broader set of phages (e.g., 12 to 24 phages from well-curated collections such as the Basel Phage Collection) should be tested.

In this revised manuscript, we have expanded the panel of phages to 12, including a selection of Basel phages chosen to represent diverse phage types including examples of Drexlerviridae (Bas01), Siphoviridae (Bas15, Bas20, Bas21), Demerecviridae (T5), Myoviridae (Bas37, T4, P1), Mosigvirus (Bas46), Vequintavirinae (Bas51), Autographiviridae (T7), and Siphoviridae (lambda). With this expanded panel we found additional defense for two retrons (retrons-Asa1 and –Squ1), but still no defense for the other two intact retrons.

**Figure 3.** A) Phage defense (log transformed) was calculated as the pfu (plaque forming units) per ml on a control strain not expressing the retron divided by pfu per ml on the retron expressing strain. Increasing numbers indicate defense phenotypes. Three biological replicates are shown as open circles and the mean is shown as a closed circle. Retron-Asa1 exhibits defense (one-way ANOVA P<0.0001) against Bas01 (P<0.0038), Bas20 (P<0.0001), Bas21 (P<0.0141), and Bas51 (P<0.0381). Individual phage statistics calculated by Dunnett’s test corrected for multiple comparisons. B) Phage defense shown exactly as in A, but for Retron-Squ1, which exhibits defense (one-way ANOVA P<0.0001) against Bas01 (P<0.0001), Bas15 (P<0.0001), Bas20 (P<0.0001), Bas21 (P<0.0001), Bas51 (P<0.0001), and T5 (P=0.0001). Individual phage statistics calculated by Dunnett’s test corrected for multiple comparisons. C) Phage defense shown exactly as in A, but for Retron-Hch1, which does not exhibit defense against this panel of phages (one-way ANOVA P=0.7125). D) Phage defense shown exactly as in A, but for Retron-Bpu1, which does not exhibit defense against this panel of phages (one-way ANOVA P=0.0644).

Additionally, it would be valuable to examine whether these retrons can provide phage defense in bacterial species other than E. coli, particularly their native hosts. This would help establish whether the protective effect is species-specific or generalizable.

This was by far the hardest for us to address, we worked hard and were able to add new compelling functional data within a natural host.

We first attempted to gain genetic access to each of these strains sourced from the environment, finding that we could transform and express genes in *Serratia quinivorans*. Next, we generated an Retron-Squ1 knockout in the natural *Serratia quinivorans* host using lambda red recombination. We tried, but were unable to obtain any previously collected phages for *Serratia quinivorans* from other researchers of phage banks. So, we attempted to isolate phages ourselves from the environment. After many attempts, we did isolate 9 phages that we could propagate on the *Serratia quinivorans* knockout strain. However, when we checked for phage defense in the wildtype strain, we found none. This is not incredibly surprising given that substantial effort has gone into creating broad and diverse phage collections for *E. coli*, whereas we had 9 uncharacterized phages to test in *S. quinivorans*. Nonetheless, we wanted to show some natural function, so we next transformed the methyltransferase from phage Bas51 that we found triggered the system in *E. coli*. In the natural host, this methyltransferase slowed the growth of the wildtype strain, but not the retron knockout strain, consistent with the abortive mechanism of retron defense. Finally, we also overexpressed the retron in the knockout background and repeated the methyltransferase experiment, finding that overexpression substantially increased the growth effect.

After establishing that these retrons are triggered by a methyltransferase in E. coli, we wondered whether they exhibit the same behavior in their native hosts. For this, we chose to examine Retron-Squ1 in Serratia quinivorans due to the genetic tractability of the host strain. We expressed the methyltransferase in both wild-type S. quinivorans (which contains a genomic copy of Retron-Squ1) and S. quinivorans with Retron-Squ1 deleted (ΔSqu1), then measured growth of the cultures over eight hours. While the wild-type strain with the methyltransferase grew similarly to an empty vector control, the ΔSqu1 strain with the methyltransferase showed reduced growth that is indicative of retron-associated toxicity (Fig 4i). Furthermore, when Retron-Squ1 borne on a high-copy plasmid was co-expressed with the methyltransferase in the ΔSqu1 strain, growth was reduced to an even greater level compared to when either plasmid was replaced with an empty vector control (Fig 4j). These results demonstrate Retron-Squ1 triggering dynamics in the context of its native host.

**Figure 4.** I) Growth curves comparing OD600 of wild-type S. quinivorans transformed with an empty vector, wild-type S. quinivorans transformed with methyltransferase, and S. quinivorans ΔSqu1 transformed with methyltransferase over 8 hrs. Methyltransferase expression was induced at 2 hrs. Bar plot on right compares OD600 measurements of these samples at the 5 hr timepoint. Open circles show each of three biological replicates and the bar shows the mean (One-way ANOVA P=0.0017; follow-up testing with Tukey’s, corrected for multiple comparisons, wt:empty vs wt:methyltransferase P=0.0045; wt:methyltransferase vs ΔSqu1:methyltransferase P=0.0021, all other conditions ns). J) Growth curves comparing OD600 of S. quinivorans ΔSqu1 transformed with either empty vector + methyltransferase, Retron-Squ1 + empty vector, or Retron-Squ1 + methyltransferase over 8 hrs. Methyltransferase expression was induced at 2 hrs. Bar plot on right compares OD600 measurements of these samples at the 5 hr timepoint. Open circles show each of three biological replicates and the bar shows the mean. (One-way ANOVA P<0.0001; follow-up testing with Tukey’s, corrected for multiple comparisons, empty&methyltransferase vs Squ1&methyltransferase P<0.0001; Squ1&empty vs Squ1&methyltransferase P<0.0001, all other conditions ns).
