# Supplementary material for: New retron systems from environmental bacteria identify triggers of anti-phage defense and expand tools for genome editing
Source: PLoS Biol. 2025 Oct 23;23(10):e3003042. doi: 10.1371/journal.pbio.3003042 (PMC12548924; doi:10.1371/journal.pbio.3003042)
Supplement: Attachment — Submitted filename: Response_to_Reviewers.docx [file pbio.3003042.s014.docx]

-----------------------------------------
REVIEWERS' COMMENTS:
-----------------------------------------

**Reviewer #1:**

*The authors have done a terrific job revising this manuscript and adding exciting additional data. I especially commend them on digging into the Bth1 retron system to identify the locus and group II intron.
I have only very modest comments on the revision:

Line 272-274: I think there is a mistake in the description of this result i.e. I believe it is the WT strain with methyltransferase that shows reduced growth whereas the retron deleted strain is unaffected?*

The reviewer is correct. We really appreciate that this error was noticed. We have fixed the text.

The wild-type strain showed reduced growth when the methyltransferase was expressed as compared to an empty vector control, and this growth effect was eliminated by deleting the endogenous Retron-Squ1 (ΔSqu1), demonstrating that this methyltransferase is able to trigger a toxic effect via the retron in its natural host (Fig 4i).

*Supplementary files 1 and 2 were not included in the manuscript link sent to me so could not be reviewed.*

Apologies, we believe that they were included. They are definitely included in this upload.  *Line 397: remove "and"?*

Removed.

**Reviewer #2:**

*I thank the authors for the very comprehensive, thoughtful, and thorough revision of their work. This is now a fantastic manuscript that will find many interested readers. All my previous comments (including quite challenging ones) have been convincingly addressed, and I have no further comments.*
